# Teacher well-being: Perceived stress, psychosocial risks, and burnout in Morocco

**DOI:** 10.1192/j.eurpsy.2024.1410

**Published:** 2024-08-27

**Authors:** H. Guider, F. Hadrya, M. A. Lafraxo, Z. Boumaaize, Y. El Madhi, A. Soulaymani, A. Mokhtari, H. Hami

**Affiliations:** ^1^Laboratory of Biology and Health, Faculty of Sciences, Ibn Tofail University, Kenitra; ^2^University Hassan First of Settat, Higher Institute of Health Sciences, Health Sciences and Technologies Laboratory, Settat; ^3^Regional Center for Education and Training Professions, Rabat, Morocco

## Abstract

**Introduction:**

Teachers encounter various challenges that can significantly affect their professional well-being. This issue stands as of paramount importance, for it not only wields influence over job satisfaction but also carries weighty implications for the quality of education provided.

**Objectives:**

This study aims to investigate the factors that affect teachers’ well-being, specifically examining the relationship between perceived stress, psychosocial risks, and burnout among public high school teachers in Tetouan, Morocco.

**Methods:**

A questionnaire survey was conducted among 258 teachers, resulting in a response rate of 57%. Three distinct instruments were used to collect data: The Perceived Stress Scale (PSS) to evaluate perceived stress levels, the Job Content Questionnaire (JCQ) to assess psychosocial risks at work, and the Maslach Burnout Inventory (MBI) for burnout measurement. We examined the bivariate correlations among these three concepts.

**Results:**

The results demonstrate significant associations among the studied factors. Perceived stress has a positive correlation with emotional exhaustion (r=0.51; p<0.01) and depersonalization (r=0.56; p<0.01), and a negative correlation with personal accomplishment (r=-0.31; p<0.01). Additionally, emotional exhaustion has a positive correlation with psychological demand (r=0.38; p<0.01). Depersonalization shows a positive correlation with psychological demand (r=0.18; p<0.05), but a negative correlation with decision latitude (r=-0.30; p<0.01) and social assistance (r=-0.24; p<0.01). Conversely, personal accomplishment presents a positive correlation with decision latitude (r=0.58; p<0.01) and social assistance (r=0.50; p<0.01).

**Conclusions:**

This study underscores the importance of decision latitude and social assistance in promoting personal accomplishment and mitigating burnout among teachers. Nonetheless, further research is required to substantiate these results and determine the fundamental cause and effect relationships.

**Disclosure of Interest:**

None Declared

